# Enhancing the Implementation of a high-quality randomized trial in pregnancy

**DOI:** 10.21203/rs.3.rs-8287988/v1

**Published:** 2026-01-12

**Authors:** Mariam Assaad, Ghada El-Hajj Fuleihan, Anwar Nassar, Sara Ajjour, Maya Rahme, Cyrus Cooper, Nicholas C Harvey, Nawal Tfaily, Taghrid Diab, Rihab Al-Tayeh, Marlene Chakhtoura

**Affiliations:** American University of Beirut Medical Center; American University of Beirut Medical Center; American University of Beirut Medical Center; American University of Beirut Medical Center; American University of Beirut Medical Center; University of Southampton; University of Southampton; Bahman Hospital; Bahman Hospital; Bahman Hospital; American University of Beirut Medical Center

## Abstract

**Background:**

Conducting clinical trials in pregnant women is essential to address pregnancy-related health issues, yet remains challenging. Hypovitaminosis D is widespread during pregnancy, particularly in the Middle East and North Africa (MENA) region. Thus, vitamin D supplementation has been suggested as a therapeutical route to alleviate the symptoms of hypovitaminosis, but its effects remain undetermined.

**Objective:**

This paper presents our experience conducting a randomized controlled trial (RCT) of vitamin D supplementation during pregnancy in Lebanon. We describe encountered challenges, strategies to address them, and key elements related to internal and external validity.

**Methods:**

We presented descriptive data derived form a double-blind randomized controlled trial in pregnant women from two centers in Lebanon. We outlined the challenges faced during the trial implementation, and our approach to address them. We detailed our strategies to ensure external validity by referencing key issues identified by Rothwell (2006). We highlighted key factors related to the internal validity of the trial using the five risk-of-bias domains from the Cochrane tool for RCTs.

**Results:**

Of 552 pregnant women screened, 60% enrolled; 88.5% completed the delivery visit, and 35.8% completed the one-month postpartum visit. Adherence to the intervention exceeded 90% in both arms. Major challenges included recruitment (35% declined participation) and center-specific dropout rates (12% vs. 7%, p = 0.004). Additional barriers included low acceptability of neonatal bone mass scans (35.8%), variability in fetal measurements, and motion artifacts in neonatal imaging. To improve external validity, we used a robust design and recruited from two centers with differing social contexts. Internal validity was strengthened through proper randomization, high adherence, minimal missing data, standardized outcome assessments, and avoidance of reporting bias. Consistent implementation and active follow-up across centers further supported study integrity.

**Conclusion:**

RCTs in pregnancy require culturally sensitive recruitment, physician engagement, and strong participant relationships. Participant-centered strategies improve adherence, reduce bias, and enhance both internal and external validity.

**Trial registration:**

clinicaltrial.gov (Trial registration number NCT02434380). URL of the registration site https//classic.clinicaltrials.gov/ct2/show/NCT02434380

## Background

Randomized controlled trials (RCTs) provide the highest level of evidence and serve as a strong foundation for systematic reviews and meta-analyses [1–3]. However, trial conduct is challenging. Indeed, recruitment and retention of study participants are the leading obstacles. Both often stem from the complexity of the trial itself, participants’ lack of awareness, understanding or trust in the research, social and cultural factors, fear of adverse events (AEs) and busy schedules [4, 5]. Lack of financial resources, unskilled project managers, poorly prepared research teams and negative publicity from media add to the staggering list [6, 7]. While these obstacles are well documented in Western countries, we are unaware of data from the Middle East North Africa region, where cultural and economic difference may present additional hurdles [8, 9]. Furthermore, trial conduct becomes a particularly challenging endeavor in pregnant women due to unique eligibility criteria within a limited timeframe, additional safety concerns with regards to the fetus, and the needed consent from another stakeholder, namely the spouse [8–15].

Hypovitaminosis D during pregnancy, a significant concern due to its potential maternal and neonatal risks, is especially prevalent in the Middle East. However, evidence on the beneficial effects of vitamin D supplementation remains inconclusive [16–21], highlighting the need for further trials in this field. This paper explores the challenges encountered in conducting an RCT on vitamin D supplementation in pregnant women and the strategies used to address them, while assessing both the external and internal validity of the conducted study.

### Objective

The primary objectives of this paper:

- Describe the key challenges encountered in implementing a randomized controlled trial during pregnancy, and the strategies used to address them.
- Outline key elements used to enhance both the internal and the external validity, thereby improving the overall quality of the trial.

## Methods

### Study Design

The Preg-D trial is a blinded randomized controlled study conducted at two sites in Beirut American university of Beirut medical center (AUBMC) and Bahman Hospital (BH) between July 2015 to June 2018. Pregnant women with serum 25-hydroxyvitamin D (25OHD) levels of 10–30 ng/ml in the early second trimester were randomized to receive either 715 IU or 2,857 IU of cholecalciferol daily until delivery (Supplementary Figure 1). The co-primary outcomes of the trial were the proportion of women achieving a desirable serum 25OHD ≥20 ng/ml at delivery and infant bone mineral content (BMC) at one month. Maternal 25OHD, fetal ultrasound measurements, and newborn parameters were assessed at key points (NCT02434380) [20].

Study preparation: Before initiating the trial, we ensured that our research question met the FINER framework (Feasible, Interesting, Novel, Ethical, Relevant) [3]. Furthermore, multiple preparatory steps were completed to enhance trial quality and ensure successful conduct, including:

- Development of a trial protocol based on SPIRIT’s 33-item checklist [22]
- Registration, and publication of the study protocol [20]
- Institutional review board IRB approval at both participating centers
- CITI certification and training for all faculty and research staff Development of recruitment strategy planning, including meetings with Obstetrics and Gynecology (OBGYN) co-investigators at both sites
- Development of Standard Operating Procedures (SOPs) and Case Report Forms (CRFs)
- Preparation of patient educational materials and clinic posters
- Coordination with pharmacists for stratified randomization and study drug preparation
- Labeling and securing of study medication by the central pharmacy
- Securing funding from WHO/EMRO (Project #EMIER1409518) and AUB
- Development of REDCap forms with validation ranges to reduce data entry errors
- Creation of study calendars and automated alerts for scheduling participant follow-ups

Adherence to study in the intervention: The adherence rate to the trial intervention was computed by dividing the number of pills that the participant took by the total number that was delivered by the study team and thus consumed for a finite study duration; Adherence was then expressed as a percentage by multiplying the result by 100.

### Challenges and solutions

We outlined the challenges encountered during the trial and the strategies implemented to address them. These challenges were assessed and addressed at various stages of the trial progression, including recruitment, screening, data collection, and participant adherence.

### External validity

We assessed the external validity of the trial using key factors suggested by Rothwell et al. (2006), (Table 1) [23].

The key factors we considered in evaluating the external validity of our study include:

1. Trial setting and study design
2. Patient selection and exclusion criteria
3. Patients’ characteristics
4. Trial protocol compared to routine practice
5. Outcome measures and follow-up procedures
6. Adverse effects reporting

### Internal validity

We explored the internal validity of the trial based on the domains described in the Cochrane risk-of-bias tool (Table 1) [24]. These include potential bias from the randomization process, deviations from the intended interventions, incomplete outcome data, biases in outcome measurement, and biases in the selection of reported results [24].

### Statistical analysis

We checked the normality of the data through histograms and stem-leaf plots. We reported categorical variables using frequencies and percentages (n, %) and continuous variables using mean ± SD. We performed the statistical analyses using SPSS version 24.0 software (IBM, Armonk, NY, USA).

We compared the characteristics of enrolled participants to those who refused enrollment, as well as participants who completed the study to those who withdrew. Acceptance and retention rates were calculated at each visit. We used t-tests and chi-square tests for continuous and categorical variables, respectively, with a p-value ≤ 0.05 considered statistically significant.

## Results

**Figure 1:**
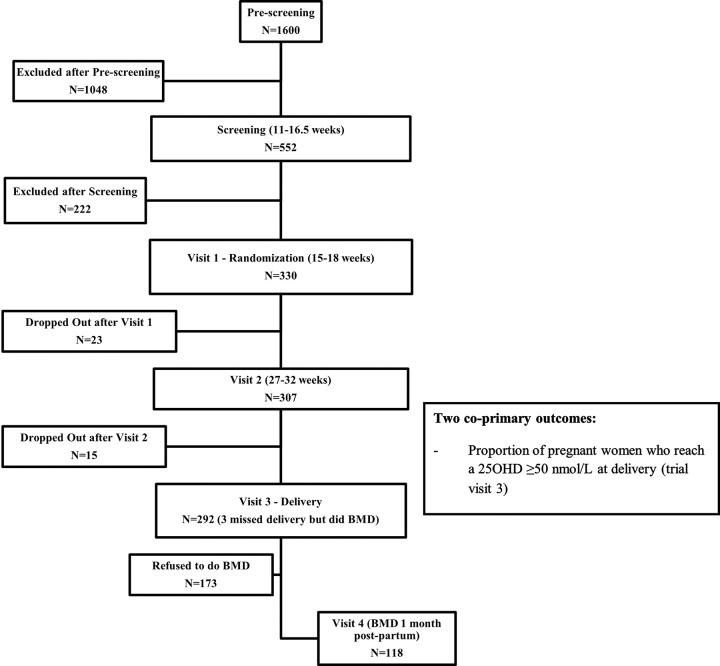
CONSORT flow diagram for the PregD Randomized Controlled Trial This figure illustrates the flow of participants through the PregD randomized controlled trial, from pre-screening to completion of all study visits. It shows the number of women assessed for eligibility, randomized, followed at each study visit, and those lost to follow-up or withdrawn at each stage. CONSORT = Consolidated Standards of Reporting Trials; PregD = Pregnancy and Vitamin D trial.

We approached 1600 pregnant women, out of which we screened 552 (34.5%). From those screened, we randomized 330 (59.8%);292 women (88.5% of those randomized) completed the study until delivery and 118 (35.8%) completed the one-month post-partum visit (Figure 1- Flow diagram).

### A. Challenges encountered during the trial implementation and our approach to address them

#### 1. Recruitment:

Recruitment was challenging, with 288 women (27.5–34.3%) declining due to disinterest or spousal objection. Other exclusions included prior vitamin D use (N=133; 12.7%), miscarriage (N=39; 3.7%), pre-screening serum 25OHD levels outside the 10–30 ng/mL range (N=50; 4.8%), and blood disorders (N=26; 2.5%). Refusal reasons varied: BH had more related to travel and miscarriage; AUBMC had more due to supplementation (Supplementary Figure 2).

To enhance recruitment, we used IRB-approved flyers, involved obstetricians, and engaged spouses when needed. At AUBMC, the OBGYN chair joined as co-investigator and promoted the study in departmental meetings. Screening referrals were made during ultrasound visits (Table 2).

#### 2. Screening and supplementation:

At screening, participants were excluded for the following reasons: 25OHD levels <10 ng/mL (N=104, 47%) or >30 ng/mL (N=36, 16%), withdrew from the study post-screening (N=68, 31%), low TSH levels (N=9, 4%), and elevated calcium levels >10 mg/dL (N=5, 2%). Notably, exclusion rates varied between centers: BH had higher exclusions due to low 25OHD levels (<10 ng/ml) (59% vs. AUBMC 36%, p=0.001), while AUBMC had a higher rate of exclusions due to elevated 25OHD levels (>30 ng/ml) compared to BH (22% vs. 10%, p=0.001). To address vitamin D deficiency, we provided supplementation and retested 25OHD levels before randomization (Table 2).

#### 3. Trial measurements:

We implemented several steps to reduce the risk of inconsistency in trial measurement. Laboratory testing for delicate hormonal studies was centralized at AUBMC to reduce variability. We developed SOPs and training materials for staff and pediatricians, particularly for the knee-to-heel measurements of neonates for the latter (Table 2). We applied identical data collection protocols to both intervention groups across four trial visits at two centers, with blinded research assistants administering surveys, recording anthropometric data, and assessing adherence.

Additionally, the IRB at AUBMC allowed only one total body scan per infant, therefore, we implemented strategies to ensure accuracy and minimize motion artifacts, such as advising mothers to breastfeed babies before scanning and trying to put babies to sleep during measurements.

#### 4. Adherence and study intervention:

In order to improve adherence to the study intervention, we implemented regular communication with participants every 2 weeks and allowed the intake of a catch-up dose according to a pre-specified protocol, emphasizing the importance of adherence to medication and study visits (Table 2). Adherence to the study intervention remained high (over 90%) across all visits. Minor differences were observed between groups, particularly at visit 3, where adherence rates were 99.4% in the high-dose group and 94.4% in the low-dose group, but these differences were not clinically significant.

#### 5. Retention and dropout:

Among the 330 participants, dropout was 11.5% (N=38), mainly between visits 1 and 2 (7%) and visits 2 and 3 (4.5%). Reasons included withdrawal (3.3%), changing gynecologist (1.8%), travel (2.1%), and abortion/fetal death (0.9%). Dropout was higher at AUBMC (16.9%) than BH (6.8%) (p = 0.004).

To improve retention (Table 2), vitamin D supplementation followed standard care, and severely deficient participants were treated before randomization. Visits aligned with routine antenatal, delivery, and pediatric care, with multiple contacts collected to reduce loss. When in-person attendance was not possible, follow-up was conducted via phone. For deliveries at non-trial hospitals, coordination was established to ensure data collection. Pediatricians addressed radiation concerns related to neonatal BMD.

Due to high refusal rates for BMD assessments (co-primary outcome), recruitment and sample size were extended with IRB approval (Table 2).

### B. Assessment of external validity

#### 1. Trial settings and study design

In our RCT trial, we established our research question a priori and detailed the study design and PICO components as part of the protocol. The target population included Lebanese pregnant women with a 25OHD level of 10–30 mg/ml. The trial interventions consisted of 2,857 IU or 715 IU of cholecalciferol daily until delivery. We had clear eligibility criteria regarding co-interventions. The outcomes were clearly defined in the protocol to ensure that the results would be widely applicable. We aligned the research questions, participant selection, measurement methods, and choice of participating centers and clinicians with real-world healthcare conditions, ensuring that the study remains applicable to routine clinical practice and generalizable across diverse patient populations. The trial was conducted in different healthcare systems, with BH representing a lower socioeconomic status (SES) compared to AUBMC, representing a higher SES, reflecting typical Lebanese pregnant women profiles, and varying healthcare needs.

#### 2. Patient selection and exclusion

Pregnant women were eligible for the study if they met the following criteria: <14 weeks gestational age (GA), of Middle Eastern origin, aged >18 years, with 25OHD levels between 10–30 ng/mL, and taking ≤200 IU/day of vitamin D. Exclusions included 25OHD <10 or >30 ng/mL, metabolic bone disease, sarcoidosis, interfering medications, >600 IU/day vitamin D, fetal anomalies, renal stones, hyperparathyroidism, uncontrolled thyroid disease, recent cancer, serum calcium >10 mg/dL, type 1 or 2 diabetes, prior gestational diabetes (GDM), vitamin D allergy, or twin pregnancy.

#### 3. Characteristics of the enrolled participants

Baseline characteristics for both randomized and non-randomized participants are presented in Table 3. Missing data were more frequent among non-randomized participants, especially for pre-pregnancy BMI (60%), veiling status (54%), and smoking status (54%), as early data collection was limited to those who were randomized. Despite this, both groups were similar across most demographic variables, supporting external validity. The only significant difference was higher alcohol use in non-randomized participants (3% vs. 0.3%, p=0.015).

Differences between recruitment centers are detailed in Supplementary Table 1. Compared to participants at AUBMC, those recruited from BH were younger (mean age 28.6 ± 5 vs. 30.6 ± 4.5 years, *p*<0.05), had lower educational attainment (17% below high school vs. 0.6%, *p*<0.005), lower spouse education levels (44.3% vs. 87% with a college degree, *p*<0.005), and lower employment rates (27.3% vs. 66.9%, *p*<0.005). Additionally, BH participants were more likely to smoke and to be veiled. This variation ensured appropriate representation from various socio-economic strata, hence enhancing generalizability.

#### 4. Trial protocol compared to routine practice

We designed the trial protocol to align with routine clinical practices and ensure external validity. Vitamin D supplementation followed typical clinical practices for pregnant women with 25OHD deficiency, based on the Institute of Medicine recommended doses [25]. Severe deficiency at screening was addressed with supplementation and repeat 25OHD measurements before randomization. In addition, we planned to have trial visits coincide with clinical visits to the obstetrician or the pediatrician.

#### 5. Outcome measures and follow-up

In our randomized controlled trial, the primary outcome was the proportion of women who achieved 25OHD levels of ≥20 ng/mL, as this serves as the most reliable indicator of vitamin D stores and holds clinical significance for both maternal and fetal health [20]. Vitamin D supplementation was provided from the early second trimester until delivery, as levels generally stabilize during the third trimester of pregnancy. Measuring BMC at 1 month is valid, supported by the previous RCTs in the USA and UK showing that maternal vitamin D supplementation during pregnancy can significantly influence neonatal BMC [21, 26]. To ensure feasibility and enhance adherence, outcome assessments in our trial were closely aligned with routine clinical care. Both maternal and neonatal measurements mirrored standard practice, and follow-up visits were scheduled to coincide with gynecological and pediatric appointments. Visit 3 procedures were timed with expected delivery dates, while neonatal BMC assessments (visit 4) were coordinated with regular pediatric visits.

#### 6. Adverse effects reporting

AEs and serious adverse events (SAEs) were monitored and reported according to the Office for Human Research Protections (OHRP)guidelines [27]. SAEs included death, life-threatening events, hospitalization, disability, congenital anomalies, or those needing medical intervention [27]. AEs were recorded via biweekly calls and assessed for clinical significance (Supplementary Table 2.A). No significant differences in AEs or SAEs were seen between dose groups.

SAEs occurred in 31 participants (9.4%), including preterm birth/NICU admission (3.3%), UTIs (0.6%), and respiratory distress (1.2%). SAE rates were comparable to those from the National Collaborative Perinatal Neonatal Network (NCPNN) at AUBMC (2015–2018), supporting generalizability (Supplementary Table 2.B). All SAEs were reviewed by the Data Safety Monitoring Board (DSMB) and deemed unlikely related to the intervention; no protocol or consent changes were needed.

### C. Assessment related to internal validity

#### 1. Randomization process

We implemented a computer-generated permuted block randomization sequence, stratified by center with a 1:1 allocation ratio. The biostatistician generated the randomization sequence and provided it to the pharmacist, who was responsible of the intervention preparation in sealed opaque boxes. This process ensured allocation concealment by using sealed intervention boxes distributed by a research assistant, thus minimizing the risk of selection bias.

#### 2. Deviations from the intended interventions

The vitamin D pills had a dose of 10.000 IU. The high dose group received 2 active pills weekly, while the low dose group took 1 active pill every 2 weeks plus matching placebos. Pills (active and placebo) were identical in appearance, taste, and smell (supplied by Europharm) to maintain blinding of participants, staff, and the biostatistician. Only the pharmacist (not involved with participants) knew the actual dosing. This approach minimized deviations from protocol. Biweekly follow-up calls supported adherence and visit attendance.

#### 3. Missing outcome data

Dropout was relatively low (11.5%), aided by strategies in Table 2. Of 330 participants, 38 dropped out before delivery: 23 (7%) between visits 1 and 2 and 15 (4.5%) between visits 2 and 3. Main reasons included withdrawal (3.3%), change of gynecologist (1.8%), travel (2.1%), and abortion/fetal death (0.9%). Rates were similar by arm (high dose: 12.7%; low dose: 10.3%; p = 0.605).

Among 292 reaching visit 3, 282 (96.6%) had serum 25(OH)D data (co-primary outcome). A total of 118 (35.8%) completed the study with neonatal BMD scans; imaging refusal was mainly due to radiation concerns. Of these, 42 scans were analyzable; 76 required imputation due to motion artifacts.

No significant differences were found between completers and dropouts in age, education, or lifestyle (Supplementary Table 3). High and low dose groups were similar, except for BMI (24.5 vs. 25.6 kg/m^2^; p = 0.019) and smoking history (33.9% vs. 44.2%; p = 0.055) (Supplementary Table 4), suggesting no meaningful imbalance or bias.

#### 4. Outcome measurement

We established (SOPs) and quality assurance protocols to ensure consistent measurements for all trial parameters, as described in Table 2, including the 2 co-primary outcomes, maternal 25OHD level and neonatal BMC. Technicians responsible for measuring 25OHD levels and neonatal bone mineral content (BMC) were unaware of the participants’ group assignments, which helped reduce potential measurement bias. Hormonal assays were centralized at AUBMC, following the Centers for Disease Control (CDC) and Decentralized Evaluation Quality Assurance System Guidance Materials (DEQAS) standards for validity. Neonatal DXA scans were performed with the Hologic Horizon A machine (version 13.5.3.1) to ensure high-quality data. Clear instructions were provided to standardize methods, and observers received training to enhance reliability. The BMD unit at AUBMC has been granted facility accreditation by the ISCD (International Society of Clinical Densitometry) in 2019 and re-certified in 2024.

#### 5. Selection of the reported result

We strictly followed the trial’s pre-specified analysis plan, as detailed in the protocol (NCT02434380), before unblinded data became available. Primary outcomes included the percentage of women reaching 25OHD ≥20 ng/ml and mean infant BMC at one month. Subgroup analysis accounted for initial 25OHD levels and season, with sensitivity analysis using Per Protocol and as-treated methods, along with adjustments for relevant variables. Both primary and secondary outcomes were clearly defined and pre-specified in the protocol before the trial began.

## Discussion

### Main Findings

The Preg-D trial identified several challenges in conducting trials in pregnant women from the Middle East. We suggest tools to address these challenges and improve the trial implementation.

### Interpretation

To address challenges related to participants’ recruitment and retention, we established strong partnerships with OBGYN colleagues, and we followed recruitment strategies during routine antenatal clinic visits. This approach, supported by prior literature, provide both effectiveness and engagement [9–11, 28–36]. A previous participant survey showed that 62% of pregnant women preferred active recruitment by their healthcare provider during clinic visits, compared to only 7% who favored passive methods (p < 0.0001) [10] [37]. Involving obstetricians in recruitment further boosted enrollment by leveraging their trusted position with pregnant women.

Dropout, a common challenge in trials, occurred at different time points in our study. 88.5% of randomized participants completing visit 3, supported by regular biweekly follow-up and collaboration with OBGYN colleagues. This retention rate aligns with other vitamin D pregnancy trials; 74–95% [21, 38–40] and non-pregnancy trials; 68–93% [41–44]. The Preg-D trial’s 11.5% dropout rate at delivery is lower than that of the MAVIDOS (15%) and Dawodu (16%) trials [21, 45]. However, the dropout increased at the neonatal DXA visit, with Preg-D at 52.4% versus MAVIDOS at 26% [21]. This was due to parents’ concerns about radiation from neonatal bone densitometry [9]. To address this, we reassured participants about minimal radiation exposure and obtained pediatrician approval and support, alleviating concerns [46]. Aligning follow-up visits with routine gynecological appointments and offering flexible rescheduling options helped maintain participant engagement and reduce dropout, consistent with prior research [9, 47–49].

We encountered challenges with spousal refusal, particularly among women from lower socioeconomic backgrounds, highlighting cultural influences. This aligns with findings from pregnancy studies conducted in Pakistan and the UAE [15, 45]. In the UAE study by Dawodu, 15% of participants discontinued either without a stated reason or due to their husband’s objection [45]. Likewise, in the Pakistani study, 38.5% of participants withdrew, with 34.4% of those cases linked to husband refusal [15]. Understanding challenges related to participant recruitment and retention is crucial for improving future RCTs. In Eastern cultures, such as in our study, the father’s approval is critical and required for pregnant women participation, unlike Western settings like the MAVIDOS study, where only one parent’s consent is required [21]. To address this, literature suggests providing separate counseling sessions for family members in Eastern contexts to reduce refusal rates and improve participation [15] [50]. Our study followed this approach by engaging husbands directly, explaining the study details, and addressing concerns, highlighting the important role of the husband’s influence in the enrollment and the retention of study participants [51]. Additionally, the MAVIDOS and SPRING trials emphasized that prior experience with research influences the willingness to participate, with those lacking such experience were more likely to decline. Addressing practical barriers and increasing research familiarity among the public could improve participation rates [52].

Building a strong rapport with the trial participants, through clear communication and engagement was key in reducing dropout and fostering commitment to the trial. Ongoing support and addressing concerns helped maintain participant’s interest and trust, as highlighted by Goldstein et al. (2021), who noted that warmth, compassion, and enthusiasm from research staff reduce anxiety and improve retention [37].

**Figure 2:**
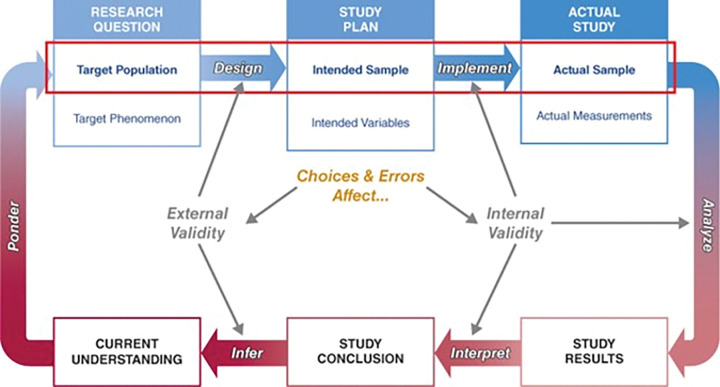
Key elements highlighting the interaction between factors affecting the external and the internal validity question This diagram illustrates the interaction between factors that shape both external and internal validity in clinical trials. It emphasizes how study design and implementation choices affect the generalizability and methodological rigor of research findings. This figure is adapted with permission from: Browner WB, Newman TB, Cummings SR, Grady DG, Huang AJ, Kanaya AM, et al. *Designing Clinical Research*. 5th ed. Philadelphia, PA: Lippincott Williams & Wilkins; 2023. Figure 3.1.

Guided by *Designing Clinical Research* by Browner et al. (2023) [3], we emphasized aligning participant selection and measurement strategies with real-world settings to enhance generalizability (Figure 2). Participants were recruited from Beirut, covering ~30% of Lebanon’s population and representing varied SES backgrounds [53]. Eligibility criteria, dosing, and outcome measures were aligned with routine care to ensure relevance and feasibility. Scheduling trial visits alongside standard obstetric and pediatric appointments further supported adherence. Maintaining internal validity was a key priority, as protocol deviations could compromise the reliability of findings. This was upheld through robust randomization with allocation concealment, strict blinding of participants and study personnel, and adherence-enhancing strategies to minimize intervention deviations. Dropout was low and similar across groups, with no major baseline differences. These efforts minimized selection, performance, and attrition biases, strengthening the study’s validity [24].

### Strengths and Limitations

This trial is one of the few studies on vitamin D during pregnancy, reflecting the difficulty of conducting trials in pregnant women in general, and more so in the Middle East, where there is a significant impact of cultural factors on participant recruitment and retention [54]. We provide lessons learnt and suggest approaches to improve trial implementation in such a particular population of interest.

We did not systematically collect reasons for non-participation. Similarly, we did not prospectively collect feedback from participants and various stakeholders on their experience throughout the trial and how its processes could have been improved. A brief optional survey could have provided valuable data to guide future engagement strategies.

## Conclusion

Conducting clinical trials in pregnant women has several challenges. Culturally sensitive recruitment methods, physician engagement, and sustained participant rapport are critical for successful trial conduct in pregnancy. Planning for participant-centered strategies improves adherence, and minimizes biases, and strengthens both internal and external validity in clinical research.

## Supplementary Material

Supplementary Files

This is a list of supplementary files associated with this preprint. Click to download.

• SupplementaryMaterial.docx

## Figures and Tables

**Table 1:** Factors that infl uence the external and the internal validity of a trial

External validity^a,b^	Internal validity^c^
Trial Setting and Study design	Randomization Process
Patient Selection and Exclusion	Deviations from the Intended Interventions
Participants’ Characteristics	Missing Outcome Data
Trial Protocol compared to Routine Practice	Outcome Measurement
Outcome Measures and Follow-Up	Selection of the Reported Result
Adverse Effects Reporting	

a Adapted from Rothwell et al 2006. ^23^

b Adapted from Designing Clinical Research, 5th Edition. Chapter 3. ^3^

c Adapted from Cochrane Handbook for Systematic Reviews of Interventions version 6.4. ^24^

**Table 2:** Challenges encountered during the trial implementation and our approach to address them

Challenge	Our approach to address the challenge

***Recruitment***	

Recruitment difficulties	Indirect recruitment model:
	• We placed IRB-approved advertisements, posters, brochures, and educational pamphlets in physicians’ clinics and prenatal-related sections.
	Direct Model:
	• We first met with all obstetricians participating in the study to explain the study rationale, review the study design and developed study procedures taking into their input consideration • We committed to recognize their contribution to the trial in authorship on all trial related primary publications • We engaged the Obstetrics department by inviting the chair as a co-investigator and by involving obstetricians to introduce potential participants to the trial. • We recruited participants face-to-face during their clinic visits at the gynecologist or prenatal ultrasound clinics and met those who expressed interest to explain the trial details. • We addressed non-participation due to husband refusal by engaging husbands directly, providing detailed explanations of the study, and responding to their concerns.
Couples refused to participate due to lack of interest or objections mostly from their husbands	
-	

***Screening and Supplementation***	
Severe vitamin D deficiency detected at screening level	• We provided vitamin D supplementation as clinically indicated in case of a 25-hydroxyvitamin D (25OHD) level less than 10 ng/ml and repeated 25OHD prior to the randomization period in case of severe deficiency.

***Trial measurements***	

Risk of inconsistency and inaccuracy in measurements	Development and Implementation of Standard Operating Procedures (SOPs) and Training: • We developed SOPs and training videos. • We used these resources to train our research team and facilitate discussions with co-investigators and collaborators • We conducted targeted training sessions for pediatricians to ensure consistent measurement of neonatal knee-to-heel length at delivery (a secondary outcome of the trial).
	Standardized Data Collection: • We used standardized methods for data collection across both centers during the four trial visits.
	Optimization of measurement settings and centralize hormonal tests • We centralized hormonal tests at AUBMC to reduce assay variability and ensure consistency in testing procedures. • We scheduled the neonatal imaging right after breast feeding when possible • We asked the mothers to try to have their baby sleep during the measurement
Neonate motion artifacts interfering with neonatal total body scan, allowed once only	

***Study Intervention and Adherence***	
Ensuring adherence to medication and compliance with study visits	Development of educational resources: • We created educational materials to enhance participants’ understanding of the intervention administration mode and adherence to trial procedures.
	Emphasize obstetrician monitoring: • We stressed the primary role of the primary obstetricians who closely followed with study participants throughout the trial.
	Ensure regular communication: • We maintained regular communication between study investigators and recruiting physicians to foster collaboration • We called participants every two weeks to encourage adherence to vitamin D intake and attendance at study visits.

***Retention and Drop-out***	

Risk of dropout	• We aligned study visits with participants’ routine antenatal and delivery appointments to minimize additional burden. • We collected multiple contact numbers from participants to enhance retention and reduce loss to follow-up. • For Visit 3 (delivery), we coordinated with participants and their physicians to ensure timely blood sample collection around the expected delivery date. • We scheduled neonatal BMD assessments during the infant’s first routine pediatric visit. • We conducted phone interviews when in-person visits were not possible. • We ensured final data collection at the time of withdrawal, by phone when necessary. For participants who dropped out close to delivery, maternal blood samples were still obtained, even participant declined neonatal BMD measurement. In cases where delivery occurred at non-trial hospitals, we coordinated with the attending physicians to collect and transfer maternal blood samples, aided by continuous communication with participants to monitor delivery timing and ensure complete data capture.
Data for participants who dropped out	

Refusal to undergo BMD assessments at visit 4	• We reassured participants about BMD related radiation, by explaining that radiation exposure as part of the trail for BMD is equivalent to about 20 hours of natural radiation, and by complying with the IRB recommendation of one attempt only for neonatal BMC measurements. • We involved pediatricians and gynecologists by asking them to personally approve neonatal bone densitometry during scheduled visits. • We complied with the IRB’s limitation of • We enrolled study subjects committed to the measurement of at least one primary outcome, 25(OH)D level or neonatal BMC*. • We extended the recruitment period and targeted a higher enrollment number in response to high dropout related to refusal of having a neonatal BMD measurement at visit 4.
High drop-out rate	

* IRB: Institutional Review Board

* BMD: Bone mineral density

* BMC: Bone Mineral Content

**Table 3.** Social, Reproductive, and Lifestyle Characteristics of Randomized Participants Compared to Non-Participants

		Not randomized (N= 222)	Randomized (N=330)	
		N (%)	N (%)	P-value
**Social Characteristics**				
**Age (years)** (Mean ±SD)		28.9 ± (5.8)	29.5 ± (4.9)	0.887
**Education (Not Rand:102, Rand:330)**	Below High School	15 (14.7)	31 (9)	0.207
	High School	18 (17.6)	49 (15)	
	College	69 (67.7)	250 (76)	
**Spouse Education** (Not Rand:100, Rand:329)	Below High School	18 (18)	60 (18)	0.608
	High School	22 (22)	58 (18)	
	College	60 (60)	211 (64)	
**Occupation (Employed)** (Not Rand:102, Rand:330)	Yes	48 (47)	151 (46)	0.818
**Reproductive Characteristics**				
**Gestational Age (weeks)** (Mean ±SD) (Not Rand:222, Rand:330)		12.7 ± (13)	12.4 ± (13)	0.032
**Parity** (Not Rand:152, Rand:330)	Nulliparous	69 (45)	132 (40)	0.166
	Primiparous	52 (34)	104 (32)	
	Multiparous	31 (21)	94 (28)	
**Gravidity** (Not Rand:152, Rand:330)	Nulligravida	0 (0)	0 (0)	0.572
	Primigravida	57 (37.5)	115 (35)	
	Multigravida	95 (62.5)	215 (65)	
**Previous Abortion** (Not Rand:151, Rand:330)	Yes	49 (32)	102 (31)	0.735
**Lifestyle**				
**BMI (kg/m^2^)** (Mean ±SD) (Not Rand:88, Rand:330)		25.1 ± (4.9)	24.9 ± (4.1)	0.79
**Active Smoker** (Not Rand:103, Rand:330)	Yes	10 (10)	39 (12)	0.555
**Have you ever smoked** (Not Rand:102, Rand:330)	Yes	46 (45)	129 (39)	0.28
**Do you drink alcohol?** (Not Rand:102, Rand:330)	Yes	3 (3)	1 (0.3)	0.015
**Did you ever drink alconol?** (Not Rand:102, Rand:330)	Yes	13 (13)	40 (12)	0.867
Do you Exercise?* (Not Rand:102, Rand:330)	Yes	13 (13)	37 (11)	0.672
**Did you Exercise?** (Not Rand:102, Rand:330)	Yes	42 (41)	128 (39)	0.666
**Veiling** (Not Rand:102, Rand:330)	Yes	60 (59)	197 (60)	0.875

Independent t-test and Chi-Square test comparing randomized subjects by center (P<0.05)

Numbers expressed as % are rounded to nearest integer

* If any type of this exercise was done: Strenuous, moderate, or gentle exercise.

## Data Availability

Data described in the manuscript, code book, and analytic code will be made available upon request pending approval.

## Abbreviations

25OHD: 25-hydroxyvitamin D
AEs: Adverse events
AUB: American University of Beirut
AUBMC: American University of Beirut Medical Center
BH: Bahman Hospital
BMC: Bone mineral content
BMD: Bone mineral density
BMI: Body mass index
CDC: Centers for Disease Control
DEQAS: Decentralized Evaluation Quality Assurance System Guidance Materials
DSMB: Data Safety Monitoring Board
IRB: Institution Review Boards
MENA: Middle East and North Africa
OBGYN: Obstetrics and Gynecology
OHRP: Office for Human Research Protections
RCTs: Randomized controlled trials
SAEs: Serious adverse events
SD: Standard deviation
SES: Socioeconomic status
SOPs: Standard Operating Procedures
SPIRIT: Standard Protocol Items Recommendations for International Trials
WHO: World Health Organization
